# Food functionality of protein isolates extracted from Yellowfin Tuna (*Thunnus albacares*) roe using alkaline solubilization and acid precipitation process

**DOI:** 10.1002/fsn3.793

**Published:** 2019-01-28

**Authors:** In Seong Yoon, Hyun Ji Lee, Sang In Kang, Sun Young Park, Young Mi Kang, Jin‐Soo Kim, Min Soo Heu

**Affiliations:** ^1^ Research Center for Industrial Development of Seafood Gyeongsang National University Tongyeong Korea; ^2^ Department of Food and Nutrition/Institute of Marine Industry Gyeongsang National University Jinju Korea; ^3^ Department of Seafood and Aquaculture Science/Institute of Marine Industry Gyeongsang National University Tongyeong Korea

**Keywords:** acid precipitation process, alkaline solubilization, fish roes, food functionality, protein isolate

## Abstract

Four types of roe protein isolates (RPIs) were prepared through the alkaline solubilization and acid precipitation (ASAP) process, and their functional properties and in vitro bioactivities were evaluated. Higher buffer capacity in pH‐shift range of 8–12 was found in RPI‐1 (pH 11/4.5), required average 94.5 mM NaOH than that of other RPIs to change the pH by 1 unit. All the samples of 1% dispersion (w/v) showed the lowest buffering capacity near the initial pH. The water‐holding capacities (WHC) of RPIs and casein as controls without pH‐shift were in range of 3.7–4.0 g/g protein, and there were no significant differences (*p *>* *0.05). At pH 2 and 8–12 with pH‐shift, WHC and protein solubility of RPIs were significantly improved compared to those of controls. Foaming capacities of RPI‐1 and RPI‐3 were 141.9% and 128.1%, respectively, but those of RPI‐2 and RPI‐4 were not detected. The oil‐in‐water emulsifying activity index of RPI‐1 and RPI‐3 was 10.0 and 8.3 m^2^/g protein, which was not statistically different from casein (7.0 m^2^/g), but lower than that of hemoglobin (19.1 m^2^/g). Overall, RPIs, casein, and hemoglobin exhibited lower food functionality at pH 4–6 near isoelectric points. Through the pH‐shift treatment, the food functionalities of RPIs were improved over the controls, especially in the pH 2 and pH 8–12 ranges. RPI also showed in vitro antioxidant and antihypertensive activities. Therefore, it has been confirmed that RPI extracted from yellowfin tuna roe has high utility as a protein‐ or food‐functional‐enhancing material or protein substitute resource for noodles, confectionery, baking, and surimi‐based products.

## 1 INTRODUCTION

According to statistics from the Food and Agriculture Organization of the United Nations (FAO), the total fishery production in 2015 was about 200 million tons (FAO, 2016). As the fish production is increasing each year, the discarding rate of fish processing by‐products also increases. Fish processing industry generates a wide variety of by‐products such as roe, visceral, heads, skin, frames, and scales in large quantities (Klomklao & Benjakul, 2016). Most of these by‐products are disposed as waste, without processing into value‐added products either for industrial applications or for animal and human consumption. However, these processing by‐products can be good protein resources (Lee, Park et al., 2016). The global demand for proteins is increasing, and more food proteins are needed from the source of conventional proteins as well as the new source of protein. If we accept that all proteins will have nutritional value, the value in the food industry for both conventional and new protein sources is required to have enough food functional properties to allow the protein to be accepted as a food ingredient (Azadian, Nasab, & Abedi, 2012; Horax, Hettiarachchy, Kannan, & Chen, 2011; Lee et al., 2017). Among fish by‐products, fish roes are highly nutritious material rich in essential fatty acids, minerals, and amino acids (Heu et al., 2006; Park et al., 2016).

Yellowfin tuna (*Thunnus albacares*) is an epipelagic fish that inhabits the mixed surface layer of the ocean above the thermocline (Kunal, Kumar, Menezes, & Meena, 2013) and is used in the canned tuna industry. It is canned with total amount of 55,135 metric tons, which accounted for 66% of total canned products in Korea (MOF, 2016). Tuna roe, a by‐product generated from fish processing (1.5%–3.0% of total weight), is generally used in animal feed or pet food preparation (Heu et al., 2006; Klomklao & Benjakul, 2016; Lee, Park et al., 2016; Lee, Lee et al., 2016). Thus, processing methods for converting the underutilized yellowfin tuna roe into more marketable and acceptable forms such as extracts, concentrates, isolates, and hydrolysates are required.

Protein modification is mostly realized by enzymatic, physical, and chemical treatment with resultant changes in structural, physicochemical, and functional properties (Gehring, Gigliotti, Moritz, Tou, & Jaczynski, 2011; Mohamed, Xia, Issoufou, & Qixing, 2012). Alkaline solubilization and acid precipitation (ASAP) process consists of isolating the protein components of fish tissue by acid or alkali and then precipitating all soluble proteins near their isoelectric points (Chaijan, Panpipat, & Benjakul, 2010; Yongsawatdigul & Park, 2004). This process allows for selective pH‐induced water solubility of tissue proteins with concurrent separation of lipids and removal of materials not intended for human consumption, such as bones, scales, and skin (Gehring et al., 2011; Tahergorabi, Beamer, Matak, & Jaczynski, 2011). The pH‐shift causes structural changes in the protein, leading to partial unfolding of proteins, thus resulting in more exposure of the functional groups (Azadian et al., 2012). The major advantages of this process include economic feasibility, high recovery yield, and improved functionality (Arfat & Benjakul, 2013). Various methods of protein isolate preparation have been reported for different protein sources, including fish protein (Azadian et al., 2012; Mohamed et al., 2012), chicken (Tahergorabi et al., 2011) and beef (Mireles DeWitt, Gomez, & James, 2002) processing by‐products, oilseeds (Horax et al., 2011), and cereals (Paraman, Hettiarachchy, Schaefer, & Beck, 2007), based on the solubility behavior of their proteins. The proteins recovered by this process have good functionality, and in some cases, better gelation properties than proteins recovered with conventional surimi processing (Chaijan et al., 2010; Kristinsson, Theodore, Demir, & Ingadottir, 2005). Protein isolates are the basic functional components of various high‐protein processed food products and thus determine the textural and nutritional properties of the foods (Mohamed, Zhu, Issoufou, & Fatmata, 2009; Mustafa, Al‐Wessali, Al‐Basha, & Al‐Amir, 1986). These properties contribute to the quality and sensory attributes of food systems.

In our earlier study, preparation of protein concentrate (Lee, Park et al., 2016; Yoon et al., 2018) and isolates (Lee, Lee et al., 2016) from tuna roe were conducted and their chemical and nutritional properties were evaluated. Also, functionalities of roe protein concentrate from tuna were examined (Park et al., 2016; Yoon et al., 2018). The aims of this study were to evaluate functional properties and in vitro antioxidant and antihypertensive activities of extracted roe protein isolates from yellowfin tuna by ASAP process for their industrial application as functional protein ingredients and supplements.

## 2 MATERIALS AND METHODS

### 2.1 Material

Yellowfin tuna (*Thunnus albacares*) roe was obtained from Dongwon F&B Co., Ltd. (Changwon, Korea). Frozen roe was partially thawed for 24 hr at 4°C and then cut into small pieces with an approximate thickness of 1.5–3 cm and minced with food grinder (SFM‐555SP, Shinil Industrial Co., Ltd., Seoul Korea). The minced roes were stored frozen at ‐20°C until the protein isolates were prepared.

### 2.2 Chemicals

Sodium dodecyl sulfate (SDS) and glycine were purchased from Bio Basic Inc., (Ontario, Canada). 2,2′‐azino‐bis(3‐ethylbenzothiazoline‐6‐sulfonic acid) diammouium salt (ABTS), hippurly‐his‐leu acetate salt (HHL), lung acetone powder from rabbit, mushroom tyrosinase, bovine serum albumin (BSA), casein, hemoglobin, sodium carbonate, sodium hydroxide, sodium L‐tartrate, and potassium hydroxide were purchased from Sigma‐Aldrich Co., LLC. (St. Louis, MO, USA). 3,4‐Dihydroxy‐L‐phenylalanine (L‐DOPA) was purchased from Acros Organics (New Jersey, USA). Copper (II) sulfate pentahydrate was purchased from Yakuri Pure Chemicals Co., Ltd. (Kyoto, Japan). Folin‐Ciocalteu's reagent was purchased Junsei Chemical Co., Ltd. (Tokyo, Japan). Soybean oil was purchased from Ottogi Co., Ltd. (Seoul, Korea). All reagents used analytical grade.

### 2.3 Preparation of roe protein isolates (RPIs)

**Figure 1:**
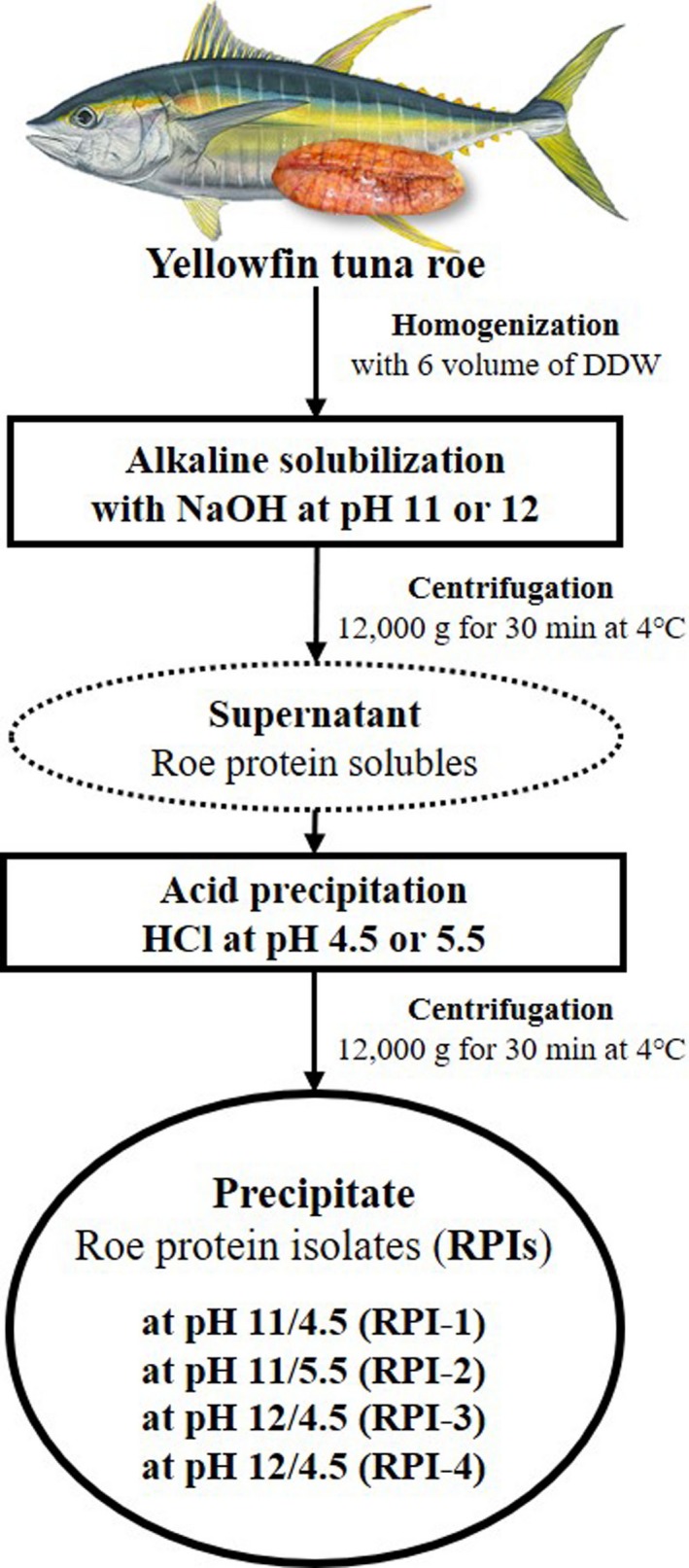
Flowchart of preparation for protein isolates from yellowfin tuna roe by alkaline solubilization and acid precipitation process

Four types of RPIs were prepared by the method of our previous report (Lee, Lee et al., 2016), and the process is shown in Figure 1. Briefly, the frozen minced roe was partially thawed and homogenized with deionized distilled water (DDW) at a ratio of 1:6 (w/v) using a homogenizer (POLYTRON^®^ PT 1200E, KINEMATICA AG, Luzern, Switzerland). The homogenate were divided into two portions and then adjusted to pH 11 and 12 with 2 *N* NaOH, respectively. Once the desired pH was reached, the alkaline solubilization was allowed to take place at 4°C for 1 hr, followed by centrifugation at 12,000 g and 4°C for 30 min using a refrigerator centrifuge (Supra 22K, Hanil Science Industrial Co., Ltd., Incheon, Korea). After centrifugation, two alkaline solubles (pH 11 and 12) in the supernatant fraction were collected. To prepare the isolates from alkaline solubles through acid precipitation, those of pH were readjusted by addition of 2 *N* HCl to pH 4.5 and 5.5, respectively, a value near the isoelectric point (pH 4‐6) of fish proteins (Chaijan et al., 2010; Pérez‐Mateos, Boyd, & Lanier, 2004; Yongsawatdigul & Park, 2004). The suspensions were centrifuged at 12,000 g and 4°C for 30 min. The precipitates by alkaline solubilization and acid precipitation (ASAP) processing were additionally washed with DDW by centrifugation at 12,000 g and 4°C for 30 min to remove the NaCl. After centrifugation, the washed roe protein isolates (RPIs) were lyophilized and referred to as RPI‐1 (pH 11/4.5), RPI‐2 (pH 11/5.5), RPI‐3 (pH 12/4.5), and RPI‐4 (pH 12/5.5), respectively. All samples were stored at ‐20°C until further experiments. Freeze‐dried concentrate (FDC) from minced roe of yellowfin tuna as a sample control was prepared using freeze dryer (PVTFD50A, ilShinbiobase Co., Ltd., Dongducheon, Korea), and casein and hemoglobin, which isolated from bovine milk and blood, respectively, as positive control were used. All experimental results were compared with the sample and positive controls.

### 2.4 Buffer capacity

Buffer capacity was estimated by the method of Park et al. (2016) with slightly modified method of Narsing Rao and Govardhana Rao (2010). Briefly, sample (300 mg) was dispersed in 30 ml of DDW and known volumes of 0.5 M NaOH or 0.5 M HCl were added and corresponding changes in pH in both alkali and acid ranges were noted. The quantity of alkali and acid added was plotted against pH. Buffer capacity in each range was expressed as the mean value of mM of NaOH or HCl per gram of protein required to bring about a change in pH by 1 unit.

### 2.5 Water‐holding capacity

The water‐holding capacity (WHC) of sample was measured following the method of Park et al. (2016). Sample (300 mg) was dispersed in 30 ml of DDW. The mixture was stirred using a magnetic stirrer at room temperature for 1 hr and then centrifuged at 12,000 g for 20 min at 4°C. Then, the supernatant was removed, and the weight of the pellet was determined.

$$\text{WHC}\left(g/g\;\text{protein}\right)=\frac{\left(\text{Weight of pellet}\;(g)-\text{Weight of sample}\;(g\right)}{\text{Weight of sample}\;(g)\times C}$$

where C is protein content (%).

### 2.6 Protein solubility

The protein solubility was measured according to the method of Park et al. (2016). Sample (300 mg) was taken in 30 ml of DDW and the pH of the mixture was adjusted to pH 2, 4, 6, 7, 8, 10, and 12 with 0.5 *N* HCl or 0.5 *N* NaOH. The mixture was stirred at room temperature (25 ± 2°C) for 30 min and centrifuged at 12,000 g for 20 min at 4°C. Protein content in the supernatant was determined using the Lowry's method (Lowry, Rosebrough, Farr, & Randall, 1951), using bovine serum albumin as a standard. Total protein content in the sample was determined using the Lowry's method after solubilization of the 20 mg sample in 0.5 *N* NaOH. Protein solubility was calculated as follows:

$$\text{Solubility}\left(\%\right)=\frac{\text{Protein content in supernatant}}{\text{Total protein content in sample}}\times 100$$

### 2.7 Foaming capacity and foam stability

Foaming capacity (FC) and foam stability (FS) of sample solution (1%, w/v) was determined according to the method of Park et al. (2016). Briefly, 10 ml of 1% (w/v) sample solution was homogenized in a 25‐ml volumetric cylinder with a homogenizer at a speed of 12,500 rpm for 1 min at room temperature. The sample was allowed to stand for 1, 15, 30, and 60 min, respectively. FC and FS were then calculated by using the following equations:

Foaming capacity (%) = VT/V_0_ × 100

Foam stability (%) = (Ft/Vt)/(VT/V_0_) × 100

where VT is total volume after whipping; V_0_ is the original total volume before whipping; and Ft and Vt are total foam and total volume after leaving at room temperature for different times (t = 15, 30 and 60 min).

### 2.8 Oil‐in‐water emulsifying activity index and emulsion stability index

The emulsifying activity index (EAI) and emulsion stability index (ESI) were determined according to the method of Park et al. (2016). Soybean oil (1 ml) and 3 ml of 1% (w/v) sample were mixed and homogenized at a speed of 12,500 rpm for 1 min. Aliquots of the emulsion (50 μl) were pipetted from the bottom of the container at 0 and 10 min after homogenization and diluted to 5 ml using 0.1% sodium dodecyl sulfate (SDS) solution. The absorbance of the diluted solution was measured at 500 nm (UV‐2900, Hitachi, Kyoto, Japan).

The absorbance measured at once (A_0_ min) and 10 min (A_10_ min) after emulsion formations was used to calculate the emulsifying activity index (EAI) and the emulsion stability index (ESI) as follows:

$$\text{EAI}\;\left(m^{2}/g\right)=\frac{2\times 2.303\times A\times D}{l\times \Phi \times C}$$

where A = absorbance (500 nm), DF = dilution factor (100), l* *= path length of cuvette (1 cm), φ = oil volume fraction (0.25), and C is protein concentration in aqueous phase (g/ml)

$$\text{ESI}\left(\text{min}\right)=\frac{A_{0}\times \Delta t}{\Delta A}$$

where A_0_ and A_10_ are the absorbance measured at once and after 10 min, ΔA = A_0_–A_10_, and Δt = 10 min, respectively.

### 2.9 ABTS^+^ radical scavenging activity

The ABTS^+^ radical scavenging activity was determined by the method of Yoon et al. (2017) with slightly modified method of Binsan et al. (2008). The stock solutions included 7.4 mM ABTS and 2.6 mM potassium persulfate. The working solution was prepared by mixing the two stock solutions in equal quantities and allowing them to react for 12 hr at room temperature in the dark. The solution was then diluted by mixing 2 ml ABTS solution with 50 ml ethanol, in order to obtain an absorbance of 1.0 ± 0.02 units at 734 nm using a spectrophotometer. Fresh ABTS^+^ ethanolic solution was prepared for each assay. Sample (1 ml) was mixed with 3 ml of ABTS^+^ solution, and the mixture was left at room temperature for 30 min in the dark. The absorbance was then measured at 734 nm using a spectrophotometer (UV‐2900, Hitachi, Kyoto, Japan). The IC_50_ value was defined as the concentration required for scavenging 50% of ABTS^+^ radical.

The absorbance measured immediately A_734_ as follows:ABTS^+^ radical scavenging activity (%) = $\frac{\left({\text{Control}}_{734}-{\text{Sample}}_{734}\right)}{{\text{Control}}_{734}}\times 100$

where control_734_ is the absorbance of same reaction system without sample.

### 2.10 Tyrosinase inhibitory activity

The tyrosinase inhibitory activity was measured by the procedure described by Iida et al. (1995) with some modification. The reaction mixture consist of 1.5 ml of 50 mM phosphate buffer (pH 6.8), 900 μl of mushroom tyrosinase (50 Unit/ml), 300 μl of sample, and 300 μl of 10 mM L‐DOPA solution. Briefly, 900 μl (50 Unit/ml of reaction mixture) of mushroom tyrosinase was preincubated with the sample in 50 mM phosphate buffer (pH 6.8) for 30 min at room temperature. Then, the 300 μl of 10 mM L‐DOPA was added to the reaction mixture and the enzyme reaction was monitored by measuring the change in absorbance at 475 nm (UV‐2900, Hitachi, Kyoto, Japan) and room temperature, corresponding to the formation of dopachrome, for 30 min at 1 min intervals.

Controls, without inhibitor, were routinely determined. The percent inhibition of the enzyme by the active compounds was calculated as follows:Tyrosinase inhibitory activity (%) = $\frac{\left({\text{Control}}_{475}-{\text{Sample}}_{475}\right)}{{\text{Control}}_{475}}\times 100$

where control_475_ is the absorbance of same reaction system without sample.

### 2.11 Angiotensin‐converting enzyme (ACE) inhibitory activity

The ACE inhibitory activity was estimated using a modification of the method of Cushman and Cheung (1971). The mixture of sample (100 μl), 50 μl of ACE extracts from rabbit lung (Sigma‐Aldrich Co., St. Louis, MO), and 50 μl of 0.05 M sodium borate buffer (pH 8.3) were preincubated at room temperature for 30 min, after which the mixture was reincubated with 50 μl of substrate (5 mM HHL in 0.05 M sodium borate buffer, pH 8.3) for 60 min at 37°C in water bath. The reaction was terminated by adding 250 μl of 1 *N* HCl. The resulting hippuric acid was extracted with 1.5 ml of ethyl acetate. After centrifugation (1,890 g, 10 min, 4°C), 1.0 ml of the upper layer was transferred into a test tube and evaporated at 100°C for 1 hr in a dry bath. The hippuric acid was dissolved in 1.0 ml of distilled water, and the absorbance was measured at 228 nm using an UV‐spectrophotometer (UV‐2900, Hitachi, Kyoto, Japan). The IC_50_ value was defined as the concentration required for inhibiting 50% of ACE.

The absorbance measured immediately A_228_ as follows:

ACE inhibitory activity (%) = $\left[1-\frac{{\text{Sample}}_{228}-\text{Control}\;{\text{Blank}}_{228}}{{\text{Control}}_{228}-\text{Control}\;{\text{Blank}}_{228}}\right]\times 100$

where sample blank is the absorbance of inactivated sample, before added HHL, and control blank is the absorbance of inactivated control, before added HHL.

### 2.12 Statistical analysis

Each measurement was replicated at least triplicates, and the results were expressed as mean ± *SD*. The data were subjected to analysis of variance (ANOVA), and the differences between means were evaluated by Duncan's test (*p* < 0.05). SPSS statistic program (SPSS 12.0 KO, SPSS Inc., Chicago, IL, USA) was used for data analysis.

## 3 RESULTS AND DISCUSSION

### 3.1 Buffer capacity

Buffer capacity is defined as mL or mmol of HCl or NaOH needed to change the pH one unit. Different nutrients in human food and animal feed increase the buffer capacity of food and feed, which is very important in human and animals. The buffer capacity and pH values of FDC, RPIs, and the positive controls (casein and hemoglobin) with pH‐shift are presented in Table 1. The initial pH values of RPI‐1 (pH 3.6) and RPI‐3 (pH 3.5) in deionized distilled water (DDW) were lower than those of RPI‐2 (pH 4.8) and RPI‐4 (pH 5.3). pH differences between RPIs are caused by a difference between the target value of pH (pH 4.5 and 5.5) in the acid precipitation of the ASAP process. The initial pH values of FDC, casein, and hemoglobin (1%, dispersion in DDW) were 5.8, 5.2, and 7.1, respectively. In the vicinity of these initial pHs, the buffer capacity of all samples (1% dispersion), including positive controls, was minimized and estimated to be near the isoelectric point. The buffer capacity of FDC was an average of 26.2 mM HCl (pH 2‐6) and 68.7 mM NaOH (pH 8‐12) for the change of one pH unit per gram of protein, respectively. For the RPIs at acidic range (pH 2–6), averages ranging from 15.0 to 28.2 mM HCl were needed per g protein for one pH unit change, whereas at alkaline range (pH 8–12), averages ranging from 74.3 to 95.7 mM NaOH were needed per g protein. Higher values for FDC may be due to presence of fat components which need more acid or alkali to bring pH change by one unit (Chalamaiah, Balaswamy, Rao, Rao, & Jyothirmayi, 2013; Lee, Lee et al., 2016). Casein and hemoglobin at pH range 2–6 required averages 18.1 and 25.0 mM HCl, respectively, to change the pH by one unit. Averages 38.6 and 29.8 mM NaOH were needed for casein and hemoglobin in the pH‐shift range 8–12, respectively. Overall, higher buffer capacities of all samples were observed for FDC, RPIs, and positive controls in alkaline pH (8–12) than in acidic pH (2–6) range. The buffer capacities of RPI‐1 and RPI‐3 were significantly better than those of other RPIs, FDCs, and positive controls (*p *<* *0.05). The buffer capacities of mrigal egg concentrate (Chalamaiah et al., 2013), gum karaya seed meal (Narsing Rao & Govardhana Rao, 2010), yellowfin tuna roe concentrate (Park et al., 2016), and skipjack tuna roe concentrate (Yoon et al., 2018) were reported to be stronger in alkaline than acidity. In these results and reports, RPIs extracted from yellowfin tuna roe were superior to those of other species through comparison of buffer capacity and were not expected to be affected by changes in external pH environment. Also, it will contribute to the design of procedures for scale‐up processing of protein isolates and hydrolysates (Narsing Rao, Prabhakara Rao, Satyanarayana, & Balaswamy, 2012; Park et al., 2016). Therefore, RPIs with excellent buffering capacity can be applied to the development of protein‐fortified food components by being applicable to various processing environments.

**Table 1:** pH and buffer capacity (accumulated mM of NaOH or HCl/g of protein) of FDC, RPIs prepared by pH‐shift process

Sample	FDC	RPI‐1	RPI‐2	RPI‐3	RPI‐4	Casein	Hemoglobin
Initial pH	5.8^b^	3.6^e^	4.8^d^	3.5^f^	5.3^c^	5.2^c^	7.1^a^
pH 2	106.5^a^	66.6^b^	107.0^a^	48.6^c^	70.5^b^	70.4^b^	100.56^a^
pH 3	41.2^b^	5.7^de^	15.9^c^	4.4^e^	13.7^c^	8.8^d^	47.88^a^
pH 4	19.1^b^	2.5^ef^	3.5 ^cd^	3.1^de^	4.1^c^	2.1^f^	20.04^a^
pH 5	6.9^c^	7.9^b^	1.2^d^	7.7^b^	0.7^de^	0.2^e^	9.60^a^
pH 6	1.4^f^	13.3^a^	5.6^c^	11.5^b^	2.7^e^	2.0^ef^	4.53^d^
pH 7	10.1^d^	24.8^ab^	11.5 ^cd^	19.6^bc^	6.8^de^	30.3^a^	0.55^e^
pH 8	36.2^b^	40.0^b^	23.8^d^	30.3^c^	14.2^e^	46.2^a^	6.99^f^
pH 9	54.9^ab^	56.2^a^	33.5 ^cd^	43.5^bc^	24.2^de^	53.4^ab^	20.52^e^
pH 10	84.6^a^	77.3^ab^	53.4 ^cd^	62.5^bc^	37.2^d^	68.0^abc^	66.99^abc^
pH 11	150.1^a^	137.3^b^	105.5^c^	130.8^b^	100.3^c^	102.6^c^	37.61^d^
pH 12	311.1^b^	418.0^a^	321.0^b^	413.7^a^	381.4^a^	200.8^c^	126.1^d^

*Notes*

Values represent the mean of *n* = 3.

Means with different small letters within same row are significantly different at *p *<* *0.05 by Duncan's multiple range test.

FDC, freeze‐dried concentrate; RPI‐1 and RPI‐2, roe protein isolate adjusting at pH 4.5 and 5.5, respectively, after alkaline solubilization at pH 11; RPI‐3 and RPI‐4, roe protein isolate adjusting at pH 4.5 and 5.5, respectively, after alkaline solubilization at pH 12.

### 3.2 Water‐holding capacity (WHC)

**Figure 2:**
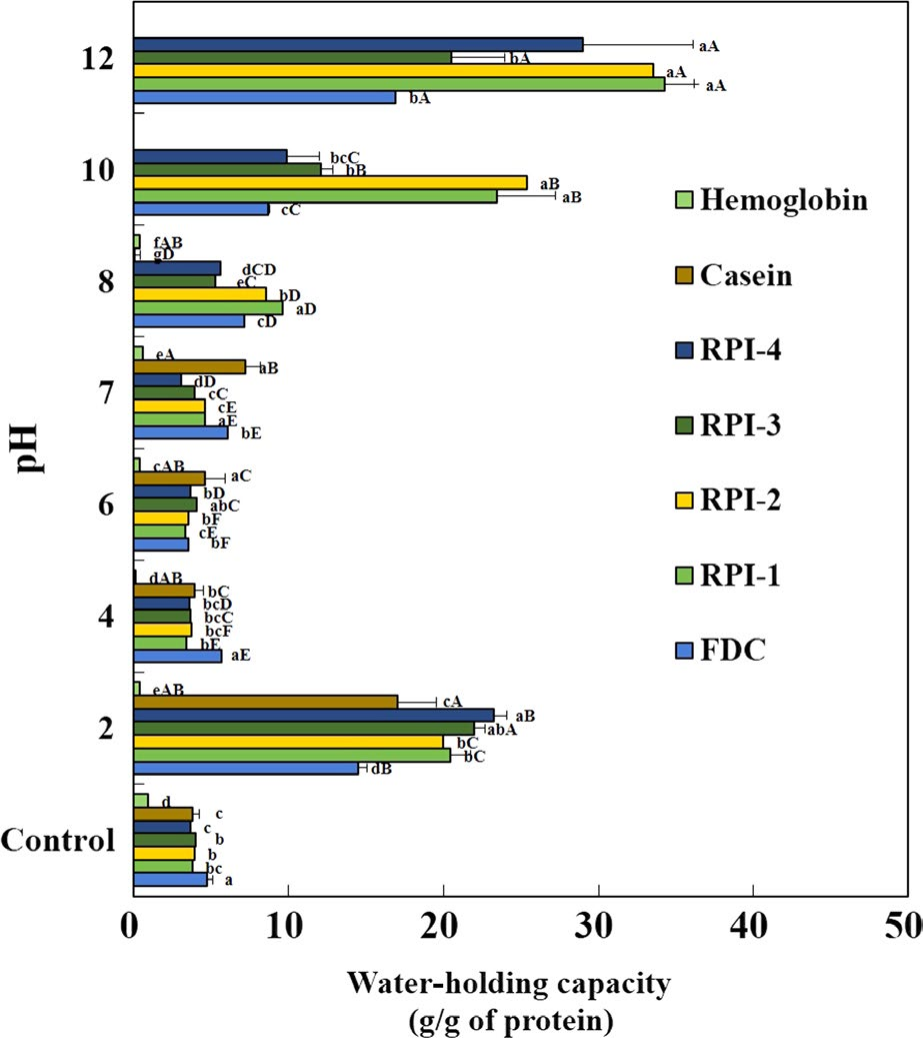
Water‐holding capacity of protein isolates recovered from yellowfin tuna roe by alkaline solublilization and acid precipitation process without and with pH‐shift. FDC, freeze‐dried concentrate; RPI‐1 and RPI‐2, roe protein isolate adjusting at pH 4.5 and 5.5, respectively, after alkaline solubilization at pH 11; RPI‐3 and RPI‐4, roe protein isolate adjusting at pH 4.5 and 5.5, respectively, after alkaline solubilization at pH 12. Values are means ± standard deviation of triplicate determinations. Means with different small letters within the same pH and capital letters within same sample are significantly different at *p* < 0.05 by Duncan's multiple range test

The WHCs (g/g protein) of FDC, RPIs, and positive controls (casein and hemoglobin) without and with pH‐shift from 2.0 to 12.0 are shown in Figure 2. Water‐holding capacity belongs to protein functionality related to hydration by protein–water interactions, and Mohamed et al. (2012) reported that protein interactions with water or oil are important in food systems because they affect the flavor and texture of the food. The WHCs of RPIs and casein without pH‐shift (controls) were in the range of 3.7–4.0 g/g protein with no significant differences (*p *>* *0.05). The WHC of hemoglobin (0.9 g/g protein) was significantly lower than those of FDC, RPIs, and casein (*p *<* *0.05). In case of pH 2, the WHC of RPIs exhibited a 20‐23 g/g of protein range, and at pH 12, showed a range of 20‐34 g/g of protein. Among the RPIs, RPI‐1 and RPI‐2 showed a relatively high water‐holding capacity. On the other hand, in the range of pH 4‐8, WHCs (3‐8 g/g protein) of RPIs were similar to the controls without pH‐shift treatment. The pH‐shift treatment significantly improved the WHC of RPIs at pH values except for the pH range of 4‐8, which minimized the water‐holding capacity due to the increased electrostatic repulsion (Azadian et al., 2012). Mohamed et al. (2012) reported that WHCs of protein isolates from tilapia were 2.63–2.51 ml/g, and lower than those of the RPIs in this study. Azadian et al. (2012) reported that the lowest WHC was observed in minced fish (pH 6.3) near the isoelectric point compared with protein isolates of silver carp. The WHC of the mrigal defatted egg protein concentrate is higher than that of the Labeo rohita fish egg protein concentrate, and the high WHC of the mrigal defatted egg protein concentrate may be due to the presence of polar groups such as COOH and NH_2_ (Chalamaiah et al., 2013). Tan, Ngoh, and Gan (2014) reported that the lack of polar amino groups on the surface of protein molecules causes WHC to be lowered because the polar groups in the protein are responsible for protein–water interactions. This is due to the acidic and alkaline pH‐shift which leads to conformational changes in the protein within the RPIs, allowing hydrophilic amino acids to easily access the surrounding water, and increase WHC.

### 3.3 Protein solubility

**Figure 3:**
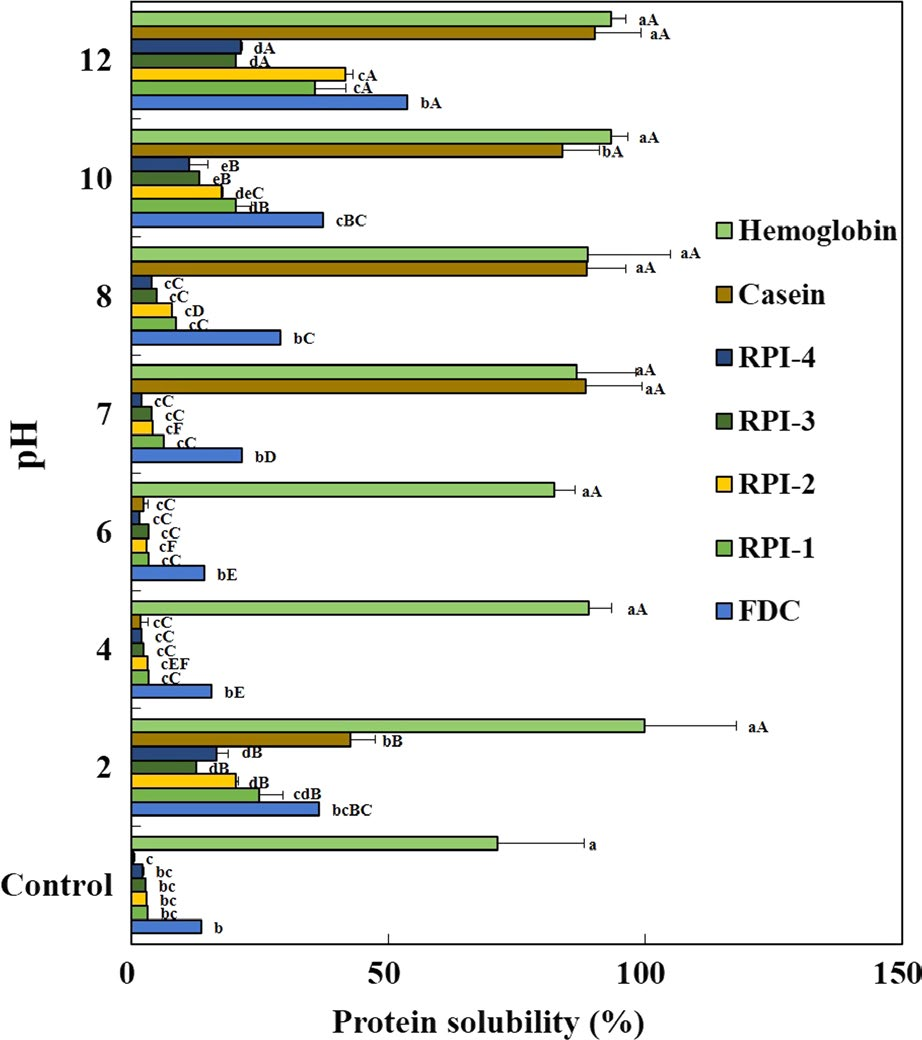
Protein solubility of protein isolates recovered from yellowfin tuna roe by alkaline solublilization and acid precipitation process at initial pH and various pH. FDC, freeze‐dried concentrate; RPI‐1 and RPI‐2, roe protein isolate adjusting at pH 4.5 and 5.5, respectively, after alkaline solubilization at pH 11; RPI‐3 and RPI‐4, roe protein isolate adjusting at pH 4.5 and 5.5, respectively, after alkaline solubilization at pH 12.Values are means ± standard deviation of triplicate determinations. Means with different small letters within the same pH and capital letters within same sample are significantly different at *p* < 0.05 by Duncan's multiple range test

The protein solubilities (%) of FDC and the RPIs without and with pH‐shift (pH 2–12) are shown in Figure 3. Protein solubility is an important parameter influencing other functionalities of proteins, such as foaming, emulsifying, and gel properties (Azadian et al., 2012; Kinsella, 1976; Mohan, Ramachandran, & Sankar, 2006). The solubilities (2.3–3.2%) of RPIs without pH‐shift (controls) were significantly lower than that (71.3%) of the hemoglobin as a positive control (*p *<* *0.05). However, the protein solubility (0.4%) of other positive control casein was found to be almost insoluble in 1% dispersion. Protein solubilities of pH‐shifted RPIs were significantly increased 12.6–24.5% at pH 2 compared to those without pH‐shift treatment (controls). Also, at pH 12, their protein solubilities ranged from 20.4 to 41.6%, indicating a higher solubility increase rate at alkaline pH‐shift. Among the RPIs at pH 12, RPI‐2 had the highest solubility (41.6%), followed by RPI‐1 (35.8%), RPI‐4 (21.3%), and RPI‐3 (20.4%; *p *<* *0.05). Also FDC (53.8%), as a sample control, showed significantly higher solubility than RPIs. Around the isoelectric point at pH 4–6, the RPIs exhibited the lowest solubility because of acid and alkali limiting protein solubilization. However, the solubility (82.3%–99.9%) of hemoglobin was not affected by pH variation, and casein showed about 90% solubility in the range of pH 7‐12. After alkaline solubilization of the ASAP process, the solubilities of protein isolates (RPI‐2 and 4) recovered in acid precipitation at pH 5.5 were significantly higher than those (RPI‐1 and 3) recovered at pH 4.5. These results indicate that extreme pH variations, such as pH 2 and 12, were able to improve protein solubility due to the exposure of more charged and polar groups to the surrounding water (Kristinsson et al., 2005). The pH‐dependent protein solubility is important in functional properties and applications related to food systems, especially at pH < 4 or > 7 (Kinsella, 1976), and is influenced by protein–protein, protein–solvent interactions, and surface hydrophobic–hydrophilic balance of the protein (Horax et al., 2011). The high solubility of fish proteins is an important feature in many food applications and affects other functional properties such as foam and emulsification properties (Kristinsson & Rasco, 2000). The protein solubilities of the RPIs extracted from yellowfin tuna roe and positive controls at various pH values may provide useful pointers on how well protein isolates will perform when incorporated into food systems (Mohamed et al., 2012).

### 3.4 Foaming capacity and foam stability

RPIs (1%, w/v) were dispersed in DDW and their foaming properties, such as foaming capacity (FC) and foam stability (FS), were analyzed (Table 2). To compare the foaming properties of solubilized protein, dispersed RPIs were centrifuged and the supernatant was analyzed for FC and FS. Before centrifugation (control 1s), the FCs (100–114.2%) of the RPIs were lower than that (121.4%) of FDC (*p *<* *0.05). RPI‐3 had the highest FC (114.2%) among the RPIs, followed by RPI‐1 (109.0%). The FCs of RPI‐2, RPI‐4, and casein were not detected. However, the FC (138.9%) of hemoglobin was significantly higher than those of FDC and the RPIs (*p *<* *0.05), because of its high solubility in DDW (Figure 3). After centrifugation (control 2s), the FCs of FDC, RPI‐1, RPI‐3, and casein were increased to 178.2%, 141.9%, 128.1%, and 109.4%, respectively, but the FCs of RPI‐2 and RPI‐4 were not still detected. The FCs of the RPIs upon pH‐shift were higher at pH 12 (166.8% for RPI‐4, 173.1% for RPI‐3, 182.9% for RPI‐1, and 199.6% for RPI‐2) than at other pH‐shift values (*p *<* *0.05). In all pH ranges, the FC of FDC ranged from 170.7% to 212.5% and was higher than those of the RPIs and positive controls (casein and hemoglobin). To show good foaming, a protein must migrate rapidly to the air–water interface, unfolding, and rearranging at the interface (Halling & Walstra, 1981; Klompong, Benjakul, Kantachote, & Shahidi, 2007). Mutilangi, Panyam, and Kilara (1996) suggested that the foaming capacity of a protein was improved by making it more flexible, exposing more hydrophobic residues, and increasing its capacity to decrease surface tension.

**Table 2:** Foaming capacity (FC, %) and foam stability (FS, %) of protein isolates recovered from yellowfin tuna roe by alkaline solublilization and acid precipitation process with pH‐shift

		FDC	RPI‐1	RPI‐2	RPI‐3	RPI‐4	Casein		Hemoglobin
Control 1	FC (%)	121.4 ± 6.3^b^	109.0 ± 3.1^bc^	100.0 ± 0.0^c^	114.2 ± 1.2^bc^	100.0 ± 0.0^c^	100.0 ± 0.0^c^		138.9 ± 18.3^a^
	15 min	88.6 ± 8.1	‐	‐	‐	‐	‐		88.6 ± 8.9
	60 min	81.5 ± 7.0	‐	‐	‐	‐	‐		81.1 ± 8.0
Control 2	FC (%)	178.2 ± 19.7^aBCD^	141.9 ± 3.7^bBC^	100.0 ± 0.0^eE^	128.1 ± 4.9^bcBCD^	100.0 ± 0.0^eC^	109.4 ± 11.1^deB^		120.9 ± 11.9^cdA^
	15 min	79.8 ± 6.9	73.1 ± 3.9	‐	53.4 ± 8.3	‐	69.3 ± 22.4		94.6 ± 5.7
	60 min	58.8 ± 4.1	‐	‐	‐	‐	‐		81.4 ± 3.4
pH 2	FC (%)	212.5 ± 13.4^aA^	132.4 ± 6.5^cdBC^	176.4 ± 6.9^bB^	131.7 ± 7.3^cdBC^	124.4 ± 6.7^cdB^	140.3 ± 23.6^cAB^		115.1 ± 8.8^dA^
	15 min	67.6 ± 6.4	68.2 ± 6.4	64.1 ± 2.5	42.6 ± 3.2	‐	73.6 ± 7.0		87.4 ± 9.7
	60 min	54.9 ± 7.4	0.0 ± 0.0	40.5 ± 2.8	‐	‐	41.3 ± 9.8		52.4 ± 12.2
pH 4	FC (%)	170.7 ± 11.6^aD^	133.6 ± 2.2^bBC^	122.4 ± 0.8^bcD^	134.3 ± 1.2^bB^	123.3 ± 0.1^bcB^	109.1 ± 10.8^cB^		125.2 ± 18.7^bcA^
	15 min	36.2 ± 1.3	70.5 ± 4.4	‐	69.7 ± .8	‐	83.4 ± 1.2		75.8 ± 8.0
	60 min	‐	‐	‐	‐	‐	73.6 ± 3.1		52.9 ± 7.5
pH 6	FC (%)	175.3 ± 0.2^aCD^	126.1 ± 2.0^bC^	127.3 ± 3.6^bCD^	124.7 ± 0.0^bCD^	100.0 ± 0.0^cC^	107.0 ± 8.4^bcB^		126.1 ± 26.5^bA^
	15 min	77.0 ± 0.8	66.7 ± 2.9	57.4 ± 1.2	‐	‐	90.3 ± 4.7		82.5 ± 8.1
	60 min	63.6 ± 0.8	‐	‐	‐	‐	76.9 ± 0.7		76.0 ± 11.5
pH 7	FC (%)	191.7 ± 2.3^aBC^	127.5 ± 3.9^cC^	121.0 ± 2.1^cD^	123.2 ± 2.1^cD^	127.2 ± 0.8^cB^	158.6 ± 37.0^bAB^		132.0 ± 17.1^bcA^
	15 min	82.9 ± 2.9	84.4 ± 0.7	82.6 ± 1.6	78.8 ± 3.8	69.6 ± 1.8	88.4 ± 3.2		82.7 ± 11.3
	60 min	69.3 ± 0.6	69.5 ± 2.2	69.3 ± 2.9	65.5 ± 0.7	55.2 ± 0.9	74.4 ± 4.5		74.5 ± 6.6
pH 8	FC (%)	180.9 ± 5.9^abBCD^	129.8 ± 1.5^cC^	133.5 ± 2.3^bcCD^	127.7 ± 4.1^cBCD^	123.7 ± 1.5^cB^	184.8 ± 65.0^aA^		139.8 ± 21.7^abcA^
	15 min	82.6 ± 3.4	84.3 ± 4.2	80.7 ± 0.5	78.5 ± 3.6	82.2 ± 3.2	65.9 ± 22.3		79.4 ± 8.4
	60 min	69.8 ± 0.2	71.2 ± 1.7	70.4 ± 0.9	67.4 ± 4.4	70.9 ± 0.1	43.1 ± 18.9		63.0 ± 5.9
pH 10	FC (%)	197.1 ± 4.7^aAB^	133.7 ± 6.7^bcBC^	129.6 ± 5.7^bcCD^	125.1 ± 1.6^bcCD^	118.9 ± 0.8^cB^	135.6 ± .8^bcAB^		140.0 ± 22.5^bA^
	15 min	71.4 ± 14.4	59.8 ± 5.3	84.7 ± 4.5	85.2 ± 3.1	84.2 ± 7.6	85.5 ± 2.6		82.2 ± 12.2
	60 min	57.1 ± 14.3	41.8 ± 5.9	65.3 ± 3.7	63.9 ± 11.9	65.3 ± 13.1	62.4 ± 1.7		56.1 ± 20.7
pH 12	FC (%)	196.4 ± 7.5^aAB^	182.9 ± 11.0^abA^	199.6 ± 10.2^aA^	173.1 ± 4.6^bA^	166.8 ± 16.3^bA^	130.5 ± 9.7^cAB^		129.7 ± 7.8^cA^
	15 min	75.1 ± 7.9	76.7 ± 9.9	84.8 ± 1.3	77.5 ± 8.8	81.1 ± 4.8	81.3 ± 11.6		84.1 ± 5.5
	60 min	63.5 ± 8.1	55.3 ± 6.1	69.7 ± 7.3	56.4 ± 11.0	61.4 ± 3.8	69.1 ± 9.1		57.1 ± 16.6

*Notes*

Controls 1 and 2 refer to samples before and after centrifugation, respectively.

Values represent the mean of triplicate determinations.

Means with different small letters within same row and capital letters within same column are significantly different at *p* < 0.05 by Duncan's multiple range test.

FDC, freeze‐dried concentrate; RPI‐1 and RPI‐2, roe protein isolate adjusting at pH 4.5 and 5.5, respectively, after alkaline solubilization at pH 11; RPI‐3 and RPI‐4, roe protein isolate adjusting at pH 4.5 and 5.5, respectively, after alkaline solubilization at pH 12; ‐, Not detected.

Prior to centrifugation (control 1s), the FSs of the RPIs were not detected because their FC was too low to keep the foam layer after whipping. After centrifugation (control 2s), FS tended to decrease with increasing time. The foams of RPI‐1 (73.1%) and RPI‐3 (53.4%) were kept for the first 15 min and then completely disappeared after 15 min. However, the foams of RPI‐2 and RPI‐4 disappeared within 15 min. The FS of RPIs with pH‐shift was more stable in the range of pH 7–12 than in the acidic range of pH 2‐6. The FSs of RPIs were unstable near the isoelectric point in the range of pH 4–6. In the range of pH 7–12, the FSs of FDC, RPIs, and positive controls were stable for 60 min. The lowest foaming capacity of all samples at pH 4‐6 is due to low water‐holding capacity (Figure 2) and solubility (Figure 3) at pH near the isoelectric point, and foam stability depends on the degree of protein–water and protein–protein interactions within foam layer (Mutilangi et al., 1996; Naqash & Nazeer, 2013). Our results also revealed that foaming properties were pH‐dependent and that the protein isolation conditions according to the ASAP process influenced foam ability (Mohamed et al., 2012).

### 3.5 Oil‐in‐water emulsifying activity index (EAI) and emulsion stability index (ESI)

The EAI and ESI were performed to assess the ability to act as emulsifiers in a variety of foods, such as soups, sauces, confectionery breads, and dairy products (Can Karaca, Low, & Nickerson, 2011). The EAI (m^2^/g of protein) estimates the ability of the protein to aid in the formation and stability of a newly created emulsion by contributing units of area of interface stabilized per unit weight of protein, which is determined by the turbidity (Park et al., 2016). The EAI (m^2^/g of protein) and ESI (min) values of FDC, RPIs, and the positive controls are shown in Tables 3. Before centrifugation (control 1s), there were no significant differences among the EAI values (2.0‐3.0 m^2^/g of protein) of the RPIs (*p *>* *0.05) except for RPI‐2 (1.4 m^2^/g of protein). The RPIs had significantly lower EAI values than that of FDC (8.3 m^2^/g of protein; *p *<* *0.05). Compared with casein and hemoglobin as the positive controls, the RPIs were higher that of casein (0.4 m^2^/g of protein), but lower than that of hemoglobin (18.4 m^2^/g of protein; *p *<* *0.05). After centrifugation (control 2s), the EAI values (4.2‐19.1 m^2^/g of protein) of the supernatants of all samples were improved compared to the dispersions before centrifugation (control 1s). The lowest EAI values of the RPIs were at pH 4 (5.4–7.7 m^2^/g of protein) with a coincidental decrease in solubility (Figure 3). Since the lowest solubility occurred at pH 4, peptides could not migrate rapidly to the interface (Klompong et al., 2007), but the EAI increased as pH moved away from pH 4. The EAI values of the RPIs were highest at pH 10, in the range of 31.1–32.5 m^2^/g of protein, except for RPI‐2. Compared to FDC, the RPIs had higher EAI values from pH 7 to 10. The EAI of casein was higher as pH increased. However, the EAI of hemoglobin (16.1–20.8 m^2^/g of protein) was similar at all pH‐shift values, except for pH 4.

**Table 3:** Emulsifying activity index (EAI) and emulsion stability index (ESI) of FDC, RPIs prepared by pH‐shift process at various pH

Sample		FDC	RPI‐1	RPI‐2	RPI‐3	RPI‐4	Casein	Hemoglobin
EAI (m^2^/g of protein)	Control 1	8.3^b^	3.0^c^	1.4^c^	2.3 ^cd^	2.0 ^cd^	0.4^d^	18.4^a^
	Control 2	12.8^bDEF^	10.0^bcDE^	4.2^dF^	8.7^cE^	6.6^cdE^	7.0^cdE^	19.1^aAB^
	pH 2	16.1^bC^	14.2^bcD^	14.8^bcE^	16.5^bD^	16.4^bD^	11.9^cCD^	20.1^aAB^
	pH 4	9.7^bF^	6.4^cdE^	5.7^dF^	5.4^dF^	7.7^cE^	2.3^eF^	12.9^aC^
	pH 6	15.9^aCD^	10.8^bD^	15.9^aE^	8.2^cE^	9.8^bcE^	9.1^bcDE^	18.1^aAB^
	pH 7	11.1^eEF^	24.5^aC^	21.7^abD^	20.0^bcC^	16.6^cdD^	13.8^deC^	18.4^bcAB^
	pH 8	13.0^dCDE^	25.5^aBC^	25.8^aC^	23.6^abB^	21.6^bC^	12.8^dC^	18.3^cAB^
	pH 10	22.2^bB^	31.2^aA^	32.5^aB^	31.7^aA^	31.1^aA^	23.7^bB^	16.1^cBC^
	pH 12	38.1^aA^	29.5^bAB^	39.2^aA^	23.7^cB^	26.4^bcB^	36.8^aA^	20.8^cA^
ESI (min)	Control 1	19.5^b^	40.2^a^	25.3^b^	41.2^a^	32.0^ab^	19.3^b^	20.0^b^
	Control 2	17.3^cCD^	23.2^bcB^	26.1^abCD^	22.9^bcD^	16.5^cC^	31.8^aB^	20.1^bcAB^
	pH 2	14.3^bcD^	17.9^abcB^	19.3^aD^	17.1^abcD^	18.3^abC^	13.2^cC^	21.2^aA^
	pH 4	14.2^cD^	32.8^abB^	45.1^aA^	29.1^abcCD^	44.6^aAB^	37.6^aB^	18.5^bcAB^
	pH 6	21.0^dABC^	63.6^aA^	45.7^abcA^	48.1^abAB^	31.7^bcdBC^	23.5^cdBC^	18.6^dAB^
	pH 7	19.5^cABC^	34.0^bB^	44.5^bA^	41.0^bBC^	61.4^aA^	14.8^cC^	18.4^cAB^
	pH 8	22.6^cAB^	24.9^cB^	40.6^bAB^	56.4^aA^	49.5^abAB^	16.0^cC^	18.3^cAB^
	pH 10	18.0^cBCD^	28.1^bcB^	33.6^bBC^	25.5^bcD^	49.4^aAB^	26.1^bcBC^	18.0^cAB^
	pH 12	24.1^bcA^	24.0^bcB^	33.3^bBC^	18.3^cD^	22.8^bcC^	62.7^aA^	16.6^cB^

*Notes*

Controls 1 and 2 refer to samples before and after centrifugation, respectively.

Values represent the mean of triplicate determinations.

Means with different small letters within same row and capital letters within same column are significantly different at *p* < 0.05 by Duncan's multiple range test.

FDC, freeze‐dried concentrate; RPI‐1 and RPI‐2, roe protein isolate adjusting at pH 4.5 and 5.5, respectively, after alkaline solubilization at pH 11; RPI‐3 and RPI‐4, roe protein isolate adjusting at pH 4.5 and 5.5, respectively, after alkaline solubilization at pH 12; ‐, Not detected.

After centrifugation (control 2s), the ESI values of the RPIs and FDC slightly decreased, except for RPI‐2, which increased to 26.1 min compared to control 1s. The ESI values of casein and hemoglobin increased to 31.8 and 20.1 min, respectively. The ESI values of the RPIs were decreased at extreme pH‐shift of pH 2 (17.1–19.3 min) and pH 12 (18.3–33.3 min). However, the ESI values of FDC and hemoglobin (14.2–24.1 min) were similar at all pH ranges. In the case of casein, the ESI was the highest at pH 12 (62.7 min). The emulsification capacity is an oil‐in‐water surface active phenomenon, which depends on the hydrophilic or hydrophobic nature of the peptides and ionic charges on particles (Chalamaiah et al., 2013; Gbogouri, Linder, Fanni, & Parmentier, 2004). The improvement in emulsification activity and emulsion stability by centrifugation is presumably due to the presence of insoluble particles in the dispersion which interferes with the formation of the emulsion layer. However, the emulsion stability did not increase in proportion to the increase in emulsifying activity according to pH rise. The large deviation of the emulsion stability by the pH‐shift treatment was presumed to be caused by the nonuniformity of emulsified particles. These results indicate that RPI‐1 and RPI‐3 are somewhat superior to RPI‐2 and RPI‐4 over the full range of pH, although there is no significant difference in the emulsifying activity and stability of RPIs by pH‐shift treatment.

### 3.6 Antioxidant and antihypertensive activity

In the above experimental results, RPI‐1 was found to be relatively superior in buffer capacity, WHC, solubility, foaming, and emulsifying ability of RPIs extracted from yellowfin tuna roe through ASAP process, and its antioxidant and antihypertensive activity were investigated. Table 4 showed the ABTS^+^ radical scavenging activity (IC_50_, μg/mL), tyrosinase inhibitory activity (%), and ACE inhibitory activity (%) of RPI‐1 (1% dispersion). Measurement of ABTS^+^ radical scavenging activity can be applied to both oleophilic and hydrophilic compounds and has been widely used as an antioxidant activity assay (You, Zhao, Cui, Zhao, & Yang, 2009). The ABTS^+^ radical scavenging activity (IC_50_) of the supernatant (1.3 mg protein/ml) of 1% RPI‐1 dispersion was 82.9 μg/ml and showed better scavenging activity than that (160‐170 μg/ml) of enzyme hydrolysates from shrimp processing by‐products (Kim, Yoon, Shim, & Lim, 2016). It also exhibited similar or slightly weaker scavenging activity than those of isolate processed water (33‐97 μg/ml, Lee et al., 2017), extracts (28‐45 μg/ml), and cooking drips (55‐110 μg/ml, Yoon et al., 2017) of fish roe.

**Table 4:** ABTS^+^ radical scavenging activity, tyrosinase inhibitory activity, and angiotensin‐converting enzyme (ACE) inhibitory activity of RPI‐1 of protein isolate recovered from yellowfin tuna roe

Sample	Protein^a^(mg/ml)	ABTS^+^(IC_50_, μg/ml)	Tyrosinase inhibitory activity (%)	ACE inhibitory activity (%)
RPI‐1^b^	1.3 ± 0.1	82.9 ± 0.9	1.4 ± 0.0	35.7 ± 2.2

*Notes*

IC_50_, the half maximal inhibitory concentration.

Values represent the mean ± *SD* of *n* = 3.

^a^Base on the Lowry's et al. (1951) methods; ^b^Supernatant of 1% dispersion after centrifugation.

Recently, tyrosinase inhibitors have become increasingly important in pharmaceutical and cosmetic products in relation to hyperpigmentation (Choi, Kim, & Lee, 2011; Schurink, van Berkel, Wichers, & Boeriu, 2007). The tyrosinase inhibitory activity of RPI‐1 was 14.0%, and some whitening effects could be expected. Tyrosinase inhibitory activities of isolate processed waters of fish roes ranged from 14.6 to 20.8% (Lee et al., 2017). Yoon et al. (2017) reported that water extracts from fish roes showed tyrosinase inhibitory activities (14.6%‐20.8%) relatively higher than those (0.4%‐2.5%) of heat‐treated cooking drips, but they were not expected to have a whitening effect. Choi et al. (2011) reported that the tyrosinase inhibitory activity of tuna cooking drip was 31%, but the activities increased in accordance with the absorbed dose of gamma irradiation. Choi et al. (2017) reported that anchovy muscle hydrolysate with subcritical water hydrolysis showed about 14.7% of tyrosinase inhibitory activity. In these experimental results and reports, tyrosinase inhibitory activity was also found in proteinous materials containing protein or amino acid, but its inhibitory activity was not strong.

The inhibition of ACE, a key enzyme regulating the blood pressure, has been recognized as the most effective therapy for the treatment of hypertension. ACE inhibitory activity of RPI‐1 (1.3 mg protein/ml) was 35.7%. Current research on natural ACE inhibition peptides has extended to seafood protein sources, particularly seafood by‐products. Lee et al. (2017) and Yoon et al. (2017) showed that the 50% ACE inhibitory activity concentration of processed waters recovered from fish roes ranged from 1.2 to 2.0 mg/ml, and these processed waters recovered through heat or alkali/acid treatment showed no difference according to treatment method in ACE inhibitory activity. On the other hand, the enzyme hydrolysates of skate skin gelatin (Ngo, Ryu, & Kim, 2014), yellow sole frame (Jung et al., 2006), skipjack roe (Intarasirisawat, Benjakul, Wu, & Visessanguan, 2013), and Pacific cod skin (Himaya, Ngo, Ryu, & Kim, 2012) showed 35%‐86% ACE inhibitory activity and similar or superior to the results of this experiment. According to these results and reports, RPIs extracted from yellowfin tuna roe showed antioxidant and antihypertensive activities and could improve these bioactivities through enzymatic hydrolysis.

## 4 CONCLUSION

The roe protein isolates recovered from yellowfin tuna contained essential amino acids‐rich proteins in our previous study (Lee, Lee et al., 2016) and had food components suitable as surimi‐based products and as protein substitutes or enhancers in traditional foods. In this study, protein isolates of yellowfin tuna roe as a processing by‐product were extracted using the ASAP process and were determined to their food functionalities and bioactivity. Yellowfin tuna roe protein isolates were similar or superior to those of the positive controls and many other fish protein isolates in terms of buffering capacity, foaming, and emulsifying ability, except for the solubility. The overall functionality of the protein isolate measured in this experiment was low at pH 4‐6 near the isoelectric point, where buffer capacity, WHC, and solubility are minimal. In addition, RPIs were also confirmed to have in vitro antioxidative and antihypertensive activities, and thus, it could be used as a health functional material. These RPIs can be used as an egg white substitute for surimi‐based products and as a development material for animal protein fortification or new agricultural and marine fusion products in snacks, noodles, confectionery, and baking. However, these protein isolates require modification to enhance their functional properties and to serve as better functional ingredients in food applications. Therefore, it is necessary to improve the solubility of RPIs through enzymatic hydrolysis for the enhancement of food and health functionalities of RPIs. This study also suggests that it would be an opportunity for the development of high value‐added products from tuna roes that are in the seafood processing industry.

## CONFLICT OF INTEREST

The authors declare no conflict of interest.

## DECLARATION

This study has nothing to do with human and animal testing.
